# Management and biosecurity practices associated with *Mycoplasma bovis* seropositivity in Swedish dairy herds: a questionnaire study

**DOI:** 10.3389/fvets.2025.1652374

**Published:** 2025-09-09

**Authors:** Karin Alvåsen, Emma Hurri, Hanna Magnusson, Madeleine Tråvén

**Affiliations:** ^1^Department of Clinical Sciences, Swedish University of Agricultural Sciences (SLU), Uppsala, Sweden; ^2^Department of Animal Health and Antimicrobial Strategies, Swedish Veterinary Agency (SVA), Uppsala, Sweden

**Keywords:** *Mycoplasmopsis bovis*, ELISA, cattle, survey, risk factors, arthritis

## Abstract

**Background and objective:**

*Mycoplasma (M.) bovis* is a significant cause of pneumonia and mastitis in cattle worldwide and is recognized for its impact on both animal welfare and farm economics. In the absence of an effective vaccine or treatment, control and prevention efforts rely on identifying risk factors associated with both within- and between-herd transmission. The aim of this study was to investigate associations between herd-level *M. bovis* seropositivity and biosecurity and management routines in Swedish dairy herds.

**Methodology:**

An online questionnaire was distributed to 146 dairy farmers in southern Sweden. The questionnaire contained 66 closed questions regarding external and internal biosecurity, calf management practices, milking routines and animal health. The targeted herds were already participating in another study, in which bulk tank milk and milk from primiparous (PP) cows were collected and analysed with IDvet ELISA to detect *M. bovis* antibodies.

**Results:**

The response rate to the questionnaire was 79% (*n* = 115) and herds were categorized as antibody negative if both bulk tank milk and samples from PP cows were negative. Of the participating herds, 31% (*n* = 36) were categorized as antibody positive as they had positive bulk tank milk and/or positive PP cows. Many farm management practices, such as purchase of cattle, were similar between *M. bovis* antibody-negative and antibody-positive herds. As a result, few management factors showed a significant association with *M. bovis* status. For external biosecurity, affiliation to the national biosecurity program (“Smittsäkrad besättning”) was associated with *M. bovis* antibody-negative status. Regarding internal biosecurity, feeding calves with milk replacer and housing weaned calves in groups of more than 15 were more common in *M. bovis* antibody-positive herds. *Mycoplasma bovis* status was also associated with animal health, as antibody-positive herds reported higher numbers of youngstock over 6 months of age that required treatment or euthanasia due to arthritis.

**Conclusion:**

These findings indicate that both internal and external biosecurity measures, including participation in a national biosecurity program and specific calf management practices, may contribute to reducing the risk of *M. bovis* infection in dairy herds.

## 1 Introduction

*Mycoplasma (M.) bovis* causes various clinical symptoms such as respiratory disease, arthritis and mastitis in cattle (1). The characteristics of this bacterium, such as its ability to evade the immune system and the lack of a cell wall, make it difficult to treat, resulting in chronic infections (2, 3). These infections have a serious impact on animal welfare and the farm economy and these should not be overlooked (4). Early detection of *M. bovis* is essential in controlling the disease. Finding the bacteria is challenging and therefore analysing antibodies is a better tool for herd-level surveillance (5). In the absence of effective vaccines, the prevention of *M. bovis* infection primarily relies on biosecurity measures. Biosecurity refers to the set of actions implemented to minimize the risk of introducing and spreading pathogens (6). The term encompasses both external biosecurity, which focuses on preventing pathogen entry, and internal biosecurity, which refers to preventing the transmission of pathogens within the herd (7). Effective control and prevention of disease transmission necessitates heightened awareness and adherence to biosecurity measures. The primary measure in a dairy herd would be to avoid introduction of *M. bovis.* This can be achieved through a closed herd policy since purchasing animals, potentially asymptomatic carriers of *M. bovis*, from other farms is a significant risk factor (8, 9). Animal contacts with other herds, including cattle shows and joint pasture, are also potential risks for *M. bovis* introduction (10). Visitors could also be a potential risk of introducing *M. bovis* to a herd. Using protective clothing and footwear were less common on *M. bovis* positive herds compared to negative herds in Finland (11). Studies have also shown that larger cattle herds have more professional visits than smaller herds, and in many cases, the visitors had direct contact with the animals (12). The risk of infection in a farm is influenced by the number of contacts with other farms, in addition to the prevalence of endemic disease (13). Other ways of transmission are connected to breeding, as viable *M. bovis* bacteria have been found in frozen semen (14) and the use of a breeding bull has been identified as a risk factor for having *M. bovis* antibodies (15).

In terms of internal biosecurity, risk factors for disease transmission within a herd include contact between infected older animals and younger calves in nearby pens (16). Shared water sources between pens, overcrowding, presence of stress factors, animal movements (cattle shows and trade), fore-stripping, and poor feed quality have also been considered as potential risk factors (10, 17). Infected milk, including colostrum, is an internal risk for disease transmission from cow to calf. It can also represent an external risk factor if colostrum is bought from another farm (18, 19). This practice, however, is uncommon among Swedish dairy herds. Internal risk factors are often associated with management practices. Key measures to prevent disease transmission include the use of dedicated hospital pens for sick animals, proper hygiene, all-in all-out practices for calves to prevent older animals infecting younger ones, culling of cows with mastitis, and avoiding overcrowding (4, 20, 21).

In Sweden, *M. bovis* occurs in dairy herds in the southern part of the country and there are large regional differences in the prevalence (3–20%) between the southern regions (22). The first case of *M. bovis* in a Swedish dairy herd was diagnosed in the most southern region in 2011 (23). Since then, the infection has spread, but the prevalence is still low compared to other European countries (15, 24, 25) which motivates actions to prevent transmission to *M. bovis*-free herds. For efficient control, knowledge about relevant risk factors is necessary. Regional variations in *M. bovis* prevalence raise questions about the role of specific management practices and biosecurity measures in disease persistence and spread. Understanding these variations is essential to tailor effective control and prevention strategies specific to Swedish dairy production. Moreover, *M. bovis* infection can increase the severity of disease in affected animals, especially in synergy with other opportunistic infections, which can have significant impacts on animal welfare and productivity (26, 27). Therefore, investigating the association between management and biosecurity practices and *M. bovis* status in Sweden is critical to supporting targeted interventions that improve disease control and herd health. Risk factors related to management and biosecurity, e.g., farm visitors, the availability of protective clothing, and animal handling procedures, are not systematically recorded in databases for Swedish cattle farms. This lack of structured data collection creates a knowledge gap, making it difficult to assess the impact of these factors on disease transmission. Consequently, it remains challenging to determine the relative importance of various biosecurity measures or to establish evidence-based recommendations for farmers. A better understanding of herd-level risk factors would enable the development of more effective strategies for preventing *M. bovis* introduction and managing infections within dairy herds. The objective of this study was to investigate associations between herd-level *M. bovis* seropositivity and biosecurity and management routines in Swedish dairy herds.

## 2 Materials and methods

### 2.1 Study population

In this cross-sectional study, dairy herds with more than 70 cows and affiliated to the Dairy Herd Improvement (DHI) program (managed by Växa Sverige) from five regions in the south of Sweden (Halland, Kalmar, Skåne, Västra Götaland and Östergötland) were targeted. These regions were chosen because cases of *M. bovis* had previously been detected there and larger herd size has been identified as a risk factor for *M. bovis* infection (22, 28). In 2020, the mean herd size in Swedish dairy herds was 98 cows (29), thus the >70 cow threshold was applied to exclude the smallest farms and focus on herds more representative of larger-scale dairy production. All respondents gave their informed consent before participating in the study, and they were informed that all data would be treated confidentially and presented in such a way that their farm identities would not be revealed. The recruitment of herds is described in detail in a previous publication (28).

### 2.2 Sampling and milk analysis

In November and December 2020, milk samples from three primiparous (PP) cows and bulk tank milk (BTM) were collected from each herd, details are described in a previous publication (28). The samples were collected in conjunction to the routine milk quality analysis at the milk testing laboratory (Eurofins Steins Laboratory, Jönköping, Sweden). The milk samples (*n* = 756, of which 145 were BTM samples and 611 individual samples from PP cows) were then sent via postal service to the Swedish University of Agricultural Sciences (SLU), Uppsala and analysed at the Department of Clinical Sciences, SLU. The analysis for IgG-antibodies to *M. bovis* was done with IDscreen^®^ indirect ELISA (IDvet, Grabels, France) according to the manufacturer’s instructions. The relative amount of antibodies in the samples was calculated as [sample optical density (OD) – negative control OD]/[positive control OD – negative control OD] × 100 (S/P%). The overnight incubation protocol was used and the cut-off S/P ≥ 30% applied.

### 2.3 Herd-level data retrieved from Växa

Mean milk production per cow in kg ECM, and the herds’ breed composition were retrieved from the DHI database for the period 1st of September 2019 to 31st of August 2020, aggregated for 12 months. A few herds (*n* = 15) were not affiliated with the DHI database, therefore breed and milk production were missing for these herds. Information on organic certification status was available for 73 herds. Data about the herd’s affiliation and status in the national biosecurity program “Smittsäkrad besättning” were retrieved from Växa Sverige. This national biosecurity program was developed by the Swedish dairy farmers’ association Växa Sverige in year 2015 to improve biosecurity on farms and provide financial compensation in the event of a salmonella standstill. Participation in the program is voluntary and structured into three levels: (1) farmers complete a self-assessment of their farm’s biosecurity and hygiene risks through an online questionnaire, followed by a biosecurity training course; (2) a veterinarian visits the farm every 18–24 months to conduct an evaluation and provide tailored advice. In addition, the herd is tested twice annually for *M. bovis* antibodies in BTM; (3) all farm employees complete an advanced biosecurity training course led by a veterinarian. There are also mandatory regulations for animal contacts and farm visitors that must be followed (30).

### 2.4 Questionnaire

A questionnaire was developed to include relevant questions about biosecurity and management practices. The survey addressed external and internal biosecurity measures, covering aspects such as herd characteristics, breeding, farm management, calf- and cow management, and animal health (Table 1). The questionnaire contained 66 closed questions and one open question; in the latter the participants had the opportunity to provide additional information or comments. The majority of the questionnaire items had previously been validated through the national biosecurity program “Smittsäkrad besättning,” as they are used in the self-assessment part of the program. The full questionnaire, translated into English, is available in the Supplementary file S1.

**Table 1:** Biosecurity routines and farm management variables in the questionnaire administered to participating dairy herds.

Questionnaire section	Variables
Farmer and herd characteristics	Job title of the answering person, number of milking cows, type of housing system
*Mycoplasma bovis*	Symptoms of *M. bovis*, possible diagnose – when and type of test
External biosecurity—animal contacts	Number of animals purchased last 12 months or last 3 years, type of bought-in animals, external contract rearing, contacts with animals from other herds, source of bought-in animals, isolation policy for bought-in animals
External biosecurity—people contacts	Number of people taking care of the animals, contacts with cattle in other herds, biosecurity routines for staff, protective clothes and boots for visitors
Breeding	Artificial insemination, own bull, insemination practices, purchased semen, use of embryos
Calving and newborn calf	Type of calving facility used, max number of cows in one group, use of calving facilities for sick animals, time for cow and calf together, colostrum feeding practices; source, time after birth, quality; assessment of quality
Calves and youngstock	Responsible person, type of milk fed, type of milk feeding equipment, cleaning and disinfection of milk feeding equipment, calf housing, location of calf facilities, sick calf housing, number of calves in each group, separation of calf groups, age of bull calves sold, surplus feed from the cows
Internal movements of animals	Number of internal transfers for calves and cows
Lactating cows	Sectioning by udder health, hygiene during milking
Animal health	Mastitis treatments, number of animals with diarrhoea, arthritis, or pneumonia

The questionnaire was constructed in the web-based service Questback Essentials (Questback Sweden AB, Stockholm, Sweden). An email with a link to the electronic questionnaire in Questback was sent to the farmers (*n* = 146) at the end of September 2020. Two reminders were sent out from the Questback program, one in October and one in November, after which the questionnaire was closed. In addition, six farmers were interviewed by phone in December 2020 (*n* = 4), January 2021 (*n* = 1) and March 2021 (*n* = 1), and the responses were entered into the online questionnaire.

### 2.5 Data management

Explanatory variables from the questionnaire were exported from the online survey tool to cvs format and were then imported to Stata/SE 18.0 (StataCorp, College Station, TX) for data cleaning and further analyses. All explanatory variables from the questionnaire were reviewed. All questions, except the last one, were of multiple-choice type and these were kept as categorical variables. The categories were recoded, when needed, and categories that contained <5% of total responses were collapsed into broader categories. Some variables were only presented descriptively and were not considered for the modelling as there was little variation in the answers. Farms having reached at least level 1 were categorised as enrolled in the national biosecurity program. One question, regarding cleaning of separate space and equipment, was excluded as there were too few responses to that question (<10%).

### 2.6 Statistical analyses

The outcome variable of interest was the herds’ *M. bovis* status from the sampling in November 2020. This outcome variable had two categories; (1) herds that had both a negative BTM sample and negative PP cows; (2) herds with positive BTM and/or positive PP cows. A directed-acyclic graph was created to identify pathways between variables to detect possible confounders (31). According to this, herd size and region were considered as possible confounders and were forced to remain in all models.

Associations between the explanatory variables from the questionnaire and the outcome (positive or negative *M. bovis* status) were first analysed in a univariable analysis using χ^2^ test for categorical variables and t-test for the two continuous variables. Univariable analyses using χ^2^ test were also done for analysing univariable associations between *M. bovis* status and reported disease outcomes. The continuous variables were assessed for the assumption of linearity with the log odds of the outcome variable. Herd size was log transformed to meet this requirement. All variables with a univariable association with the outcome of *p* < 0.2 were included in the multivariable logistic regression model (Table 2). Variables with an association with the outcome of *p* > 0.2 are found in Supplementary Table S2. Collinearity between the explanatory variables was assessed with a variance inflation factor, where a value larger than 10 was considered to indicate significant collinearity between explanatory variables (32). The questions about the introduction of new animals to the farm were highly collinear and the variable with the strongest univariable association with the outcome was used in the multivariable models.

**Table 2:** Distribution of potential risk factors associated with having a negative (n = 79) or positive (*n* = 36) *M. bovis* status subsequently included in the multivariable logistic regression models.

Variable	Category	*M. bovis* status		*P*-value
		Negative herds, n	Positive herds, n	
External risk factors				
Enrolled in biosecurity program	No	18	30	<0.001
	Yes	61	6	
General time period for keeping new animals separately	< 2 weeks	17	2	0.064
	2 to 4 weeks	14	11	
	> 4 weeks	12	9	
	Not applicable	36	14	
Introduction of new animals	No purchases	54	17	0.096
	From 1 farm	13	10	
	From >1 farm	12	9	
Number of animals introduced	None	53	17	0.101
	1 to 5	8	7	
	>5	17	12	
Separate care of purchased animals	Yes	41	25	0.134
	No	9	1	
	Not applicable	29	10	
Use of external contract rearing for heifers	No	75	31	0.102
	Yes	4	5	
Internal risk factors				
Calves have their own teat bucket	No	33	8	0.038
	Yes	45	28	
Colostrum feeding to calves is provided by	Teat bottle	20	18	0.027
	Suckling with supervision	16	5	
	Teat bucket	26	6	
	Suckling (no supervision)	12	2	
	Tube feeding	5	5	
Grouping of cows by udder health status	Always	16	10	0.100
	Sometimes	13	1	
	Never	49	25	
Milk-fed calves are offered milk replacer (powder)	No	54	15	0.005
	Yes	24	21	
Number of milk-fed calves per group	2 to 8	53	22	0.148
	>9	18	14	
Number of weaned calves per group	3 to 8	39	10	0.000
	9 to 15	34	12	
	>15	6	14	
Separation of cow and calf after birth is performed	Immediately	10	10	0.096
	Within 12 h	12	8	
	12 to 24 h	32	12	
	After more than one day	25	6	
Testing the antibody content in the colostrum	Never done	32	13	0.003
	Sometimes	26	3	
	Always	21	20	
Use of sick pen for isolation of unhealthy calves	In most cases	25	18	0.068
	Sometimes	39	16	
	No	15	2	
Weaned calves are housed	In separate building or outside	40	17	0.160
	With dairy cows	11	1	
	With dry or fresh cows	13	6	
	With other youngstock	15	12	
Where are the milk-fed calves kept	Separate calf barn	27	16	0.034
	Together with lactating cows	17	2	
	With dry cows, fresh cows and/or young stock	20	7	
	Outside	9	10	
Confounders				
Region	Skåne	14	11	0.340
	Halland	16	5	
	Kalmar/Kronoberg	9	6	
	Västergötland	31	9	
	Östergötland	9	5	
Herd size^a^		154 (SD = 127)	257 (SD = 198)	0.001

a Number of dairy cows: reported as mean and standard deviation (SD).

As the initial full model did not converge, all variables with univariable association with the outcome of *p* < 0.2, were divided into sub-models for internal and external risk factors, respectively. The variables included in the respective models are indicated in Table 2. Logistic regression models were fitted, in which region and herd size were forced in as confounders in both models. A manual backwards elimination procedure was used in which the variables with the largest *p*-values were subsequently removed while evaluating changes in coefficients of the retained variables. This process continued until all remaining effects had a p-value of less than 0.05.

To address missing values in the internal risk factor analysis, multiple imputation was applied to the dataset. This method increases the number of observations available for analysis, reducing bias and improving statistical power (33). By minimizing variability across variables, multiple imputation allowed for a more comprehensive inclusion of farms in the multivariable model compared with a complete-case analysis. Before performing imputation, we assessed the missing data pattern using the ‘*misstable patterns*’ command in Stata to verify its suitability for multiple imputation. We conducted 20 imputations over 20 iterations, with default settings for other parameters. Variables associated with the outcome at *p* < 0.2 in the univariable analysis were included in the imputation process. We applied logistic regression imputation for binary data, multinomial regression imputation for unordered categorical data, and proportional odds model for ordered factors with ≥2 levels. To evaluate the plausibility of the imputed values, we plotted imputed and observed values across iterations. The fit of the multivariable models were assessed by Hosmer-Lemeshow goodness-of-fit tests and examined graphically using deviance and Pearson residuals for potential outliers.

## 3 Results

### 3.1 Study herds

The survey targeted 146 herds participating in the longitudinal study by Hurri et al. (28) in September 2020 when the questionnaire was sent out. The final dataset included 115 herds (response rate, 79%), including 79 and 36 herds with negative and positive *M. bovis* status, respectively. Of the 36 farms classified as having a positive *M. bovis* status, 23 had both a positive BTM sample and positive PP cows, 12 farms had a negative BTM sample and positive PP cows, and one farm had a positive BTM sample and negative PP cows. For the included herds, the median herd average milk production per cow in year 2019/2020 was 11,076 kg ECM (Q1 = 10,357, Q3 = 11,817) and the median herd size was 135 cows (Q1 = 84, Q3 = 220). Of the 73 herds for which data were available, 12 were certified for organic production. Of these 12 organic herds, all but one had a negative *M. bovis* status. Indicating a trend toward negative *M. bovis* status in organic herds, however, this association was not statistically significant (*p* = 0.07, Pearson χ^2^). On most farms (*n* = 98), the questionnaire was completed by the farm owner. None of the herds had imported cattle from abroad over the last five years. Nearly all farms (*n* = 111) provided at least one set of protective clothing and boots for visitors. The four farms that did not offer protective clothing and boots were all part of the *M. bovis-*negative group.

### 3.2 Management practices associated with *M. bovis* status

All tested variables considered as potential risk factors for a positive *M. bovis* status are presented in Table 2 and in Supplementary Table S2, along with descriptive statistics and univariable associations with herd *M. bovis* status. The final multivariable logistic regression model for external risk factors is presented in Table 3. The results indicate that enrolment in the national biosecurity program “Smittsäkrad besättning” was significantly associated with having a negative *M. bovis* status. The confounding variable ‘herd size’ was significant in this model, demonstrating an increased likelihood of a positive *M. bovis* status as herd size increased.

**Table 3:** Final model for external risk factors associated with having a positive *M. bovis* status (*n* = 36) compared with being a herd with a negative *M. bovis* status (*n* = 79).

Variable	Odds ratio	SE^a^	*P*-value	95% CI^b^
Herd size^c^^,^^d^	2.63	1.05	0.02	1.20; 5.73
Region^d^				
Skåne	Referent			
Halland	0.22	0.19	0.08	0.04; 1.22
Kalmar/Kronoberg	0.71	0.63	0.70	0.12; 4.03
Västergötland	0.38	0.28	0.19	0.09; 1.60
Östergötland	0.39	0.35	0.29	0.07; 2.27
Enrolled in biosecurity program				
No	Referent			
Yes	0.05	0.03	0.00	0.01; 0.15
Intercept	0.03	0.06		

a SE, standard error.

b CI, confidence interval.

c Log-transformed average herd size.

d Included as confounder.

The final model with imputed data for internal risk factors (Table 4) showed that farms feeding milk replacer to their calves and farms that kept weaned calves in larger groups than 15 calves had higher odds of a positive *M. bovis* status. The complete-case analysis (Supplementary Table S3) and the imputed data analysis for internal risk factors identified the same two significant variables, with only minor differences in coefficient estimates.

**Table 4:** Final model on imputed data for internal risk factors associated with having a positive *M. bovis* status (*n* = 36) compared with being a herd with a negative *M. bovis* status (*n* = 79).

Variable	Odds ratio	SE^a^	*P*-value	95% CI^b^
Herd size^c^^,^^d^	1.95	0.81	0.11	0.86; 4.39
Region^d^				
Skåne	Referent			
Halland	0.50	0.37	0.35	0.11; 2.15
Kalmar/Kronoberg	1.03	0.82	0.97	0.22; 4.91
Västergötland	0.45	0.28	0.20	0.13; 1.53
Östergötland	0.84	0.64	0.81	0.18; 3.78
Use of milk replacer for calves				
No	Referent			
Yes	3.46	1.78	0.02	1.26; 9.49
Group size of weaned calves				
3 to 8	Referent			
9 to 15	0.75	0.42	0.61	0.25; 2.28
>15	4.75	3.43	0.03	1.15; 19.6
Intercept	0.01	0.02		

a SE, standard error.

b CI, confidence interval.

c Log-transformed average herd size.

d Included as confounder.

For all models, the Hosmer-Lemeshow goodness-of-fit test was not significant, indicating an adequate fit.

### 3.3 Animal health outcomes

One section of the questionnaire included farmers’ reported disease outcomes in their herds. Herds with a positive *M. bovis* status had more youngstock over 6 months of age treated or euthanized due to arthritis (Table 5). No statistically significant univariable associations were identified between *M. bovis* status and any of the other disease outcomes, including mastitis treatments in cows and respiratory disease in calves.

**Table 5:** Disease outcomes reported by farmers in herds with antibody-negative (*n* = 79) or antibody-positive (*n* = 36) *M. bovis* status.

Variable	Category	Negative herds	Positive herds	*P*-value
Mastitis treatments	<5%	25	8	0.435
	5–10%	28	15	
	10–15%	17	11	
	>15%	9	2	
Calf diarrhoea	None/few	38	15	0.369
	around 25%	28	17	
	around 50%	12	3	
Calves treated for respiratory disease	None/few	61	24	0.189
	Around 25%	17	12	
Calves treated or euthanized due to arthritis	None	51	18	0.094
	1 to 8	24	17	
Youngstock over 6 months treated or euthanized due to arthritis	None	61	20	0.002
	1 to 6	11	15	
Cows treated or euthanized due to arthritis	None	47	14	0.061
	1 to 15	29	19	

## 4 Discussion

This study explored herd-level factors associated with *M. bovis* antibody status in Swedish dairy farms, emphasizing the role of external and internal biosecurity. In this study, 31% of the herds were classified as antibody-positive, compared to the national screening in 2019, where 8% (range: 3 to 20%) tested positive in BTM in these regions (22). This discrepancy is partly due to our inclusion of test results from PP cows in the classification of *M. bovis* status. Farm management practices including the history of purchasing cattle to the farm were similar between antibody-positive and antibody-negative farms. Therefore, few management factors that had a statistically significant association with the herd *M. bovis* status were identified. Our findings demonstrated that participation in the national biosecurity program “Smittsäkrad besättning” was significantly associated with having a *M. bovis* antibody-negative status. Additionally, the internal management practices of feeding milk replacer to the calves and housing weaned calves in large groups (>15) were more common in *M. bovis* antibody-positive herds. These results highlight the importance of both external and internal biosecurity measures in preventing disease introduction and transmission within dairy herds.

### 4.1 External biosecurity practices associated with *M. bovis* status

The protective effect of being enrolled in a biosecurity program aligns with previous research emphasizing the role of biosecurity practices in reducing infectious disease transmission (6, 34). The “Smittsäkrad besättning” national biosecurity program mandates specific hygiene measures, including the use of protective clothing and boots for visitors, a designated visitor entrance, and a hygiene station with soap and water. Furthermore, enrolled herds must be kept separate from non-enrolled cattle farms. Of the farms categorized as enrolled in the national biosecurity program in the analysis, all but one had completed level 2, meaning they had received at least one veterinary visit for evaluation and discussion of biosecurity routines and hygiene standards. One farm had only reached level 1, which involved self-evaluation and biosecurity information. Adherence to the national biosecurity program’s regulations, coupled with increased biosecurity awareness, is expected to reduce the risk of infectious disease introduction. Our study supports this hypothesis, demonstrating that participation in the program is associated with having a negative status.

A majority of the farms (n = 67) did not introduce new cattle or reintroduce returning animals during the past 12 months. Widgren and Frössling (35) analysed cattle movements in Sweden over a 3.5-year period and found that both short- and long-distance transports occurred on a weekly basis. Despite this, the majority of cattle (75%) remained on a single holding throughout the 3.5 years period, while 23% were transferred to a different holding once. Similarly, Nöremark et al. (36) investigated contact networks among livestock farms and reported that most holdings had few or no connections, whereas a smaller subset exhibited high connectivity with other farms. While maintaining a closed herd is an important biosecurity measure, its practical implementation in dairy herds can be challenging. Farms may want to introduce new animals if internal recruitment of youngstock is insufficient, or if the farm is expanding and wants to rapidly get the stalls fully occupied (37–39). The introduction of animals with uncertain health status poses a risk for pathogen transmission (9, 40). Introducing new animals, including a breeding bull, is considered the most important risk factor for introducing *M. bovis* into a herd (15, 41). When introducing new animals to the herd, biosecurity measures related to keeping the new animals separated from the other cattle on the farm, were always implemented on 44 farms and sometimes implemented on 23 farms, with similar practices observed in both antibody-positive and antibody-negative herds. In another study (28), the introduction of animals was associated with higher antibody levels in PP cows; however, we did not find a correlation on herd-level in the current study. It is possible that the limited number of herds in this study, coupled with that some of the herds may have been infected with *M. bovis* for several years, may have influenced the results. The antibody-positive status in BTM may remain consistent over at least 2 years, possibly longer (28).

The study also confirmed that herd size was a significant confounder, with larger herds more likely to have a positive *M. bovis* status. This finding is consistent with previous reports suggesting that increased herd size is associated with higher risk of being *M. bovis* positive (9, 22, 42). Larger and expanding herds have been reported to experience greater within-herd animal movement, increased contact between calves and adult cattle, and higher labor turnover, potentially introducing biosecurity lapses (24). On the other hand, larger herds may have better opportunities to implement stricter biosecurity measures, as they can allocate specific personnel to manage different animal groups, thereby reducing contact between groups within the farm (43, 44). Although this study did not directly identify any labor-related biosecurity risks, such factors may have been mitigated or obscured by participation in the national biosecurity program, which includes training and regular evaluation of farm biosecurity measures. The herds included in this study had not reached level 3 of the national biosecurity program, which involves biosecurity training for employees. Future studies should investigate the impact of staff biosecurity training and adherence to farm protocols.

### 4.2 Internal biosecurity practices associated with *M. bovis* status

For the internal biosecurity, feeding calves with milk replacer was more common in *M. bovis* antibody-positive herds. There is a risk of *M. bovis* transmission from cows to calves through infected milk (1, 19). Therefore, feeding milk replacer to calves is a general recommendation for *M. bovis* positive herds to minimize this risk and may thus be a consequence of the herd status rather than a risk factor for introduction of *M. bovis*. Also, the use of milk replacer might be associated with the production system, a potential confounder since organic farming tended to be associated with a negative *M. bovis* status. In organic farming, calves are reared on whole milk, for at least 12 weeks (45), whereas conventional systems typically allow a minimum milk-feeding period of 8 weeks, with milk replacer as a common feed source (46). Certified organic farms are restricted to purchasing animals exclusively from other organic farms, thereby limiting external herd contacts (45). This may reduce the risk of introducing infections as shown by Bidokhti et al. (47). Unfortunately, information on the production system was only available for 73 herds, and it was therefore not possible to differentiate conventional dairy farms from those operating under organic production in the regression analyses. To clarify the potential impact of production systems on management practices and *M. bovis* status, we recommend future studies to include this variable.

Furthermore, we found that housing weaned calves in groups larger than 15 calves was associated with *M. bovis* antibody-positive herd status. This result could possibly be linked to an increased number of animal-to-animal contacts and amplified pathogen circulation within the group and within the herd. Smaller calf groups (3–9 calves) have been shown to reduce respiratory disease and increase growth rate in young dairy calves compared to calves reared in larger groups (48, 49). *M. bovis* can be transmitted within-herd both from calves to cows and from cows to calves (50). Another study reported that *M. bovis* infection spread to all age groups after a mastitis case (51). Young clinically healthy animals had prolonged colonisation in nasal discharge and were suggested to serve as a reservoir of *M. bovis* with continued circulation (10). Therefore, to reduce disease spread it is important to break the infection route among the calves, and small groups without contact with older animals is a recommendation (11).

### 4.3 Animal health

The number of arthritis cases treated or euthanized in youngstock were higher in *M. bovis* positive herds in this study, with a tendency for a higher frequency also in calves and cows. *M. bovis*-associated arthritis tends to be sporadic, although outbreaks can occur (1). Animals with *M. bovis* arthritis become acutely lame, displaying swollen joints and tendon sheaths, and respond poorly to antibiotic treatment resulting in culling of the animals (52, 53). Considering this, arthritis is both an animal welfare issue and has an economic impact on the farm. Some years ago, arthritis was also the most common form of *M. bovis*-associated disease in Danish farms with a recent outbreak (54). This study is the first to highlight that arthritis might be a problem for *M. bovis* positive Swedish dairy herds. The number of arthritis cases is difficult to access through DHI data (Växa Sverige) as it is aggregated under lameness disorders along with claw diseases (22, 28). Lameness was localized to the claw in more than 82% of the cases in a North American study (55) and it is believed to be similar in Sweden. Since the number of arthritis cases in this study was reported by the farmers, it may be biased, as farmers vary in their ability to detect problems and make accurate diagnoses. We speculate that the lack of observed differences in other health parameters between *M. bovis* positive and negative herds may be due to the limited contribution of *M. bovis* to the overall disease burden. Although *M. bovis* is associated with mastitis it typically accounts for only a small proportion of mastitis cases, and udder health is influenced by multiple other factors, including milking hygiene, environmental conditions, and the presence of other pathogens. Similarly, respiratory disease in dairy cattle is multifactorial, and the presence of *M. bovis* alone may not be sufficient to result in a detectable increase in disease prevalence at the herd level.

### 4.4 Modelling considerations

Multiple imputation is a flexible, simulation-based statistical technique for handling missing data and has been shown to generally produce less bias than complete-case analysis (56). In this study, both the complete-case analysis and the analysis of the imputed dataset resulted in the same variables being retained in the final models. The imputation process increased the number of usable observations from 105 in the complete-case analysis to 115. Including data from these additional ten farms likely contributed to reducing bias in the study results.

Internal and external risk factors were analysed separately. The interaction between external and internal biosecurity measures warrants further investigation. Farms with robust external biosecurity measures might require less stringent internal control due to a lower risk of pathogen introduction. In contrast, farms that have already contracted *M. bovis* may need to implement stricter internal measures to control the disease, limit its impact and prevent transmission to uninfected animals. Due to the large number of variables, interaction effects between internal and external biosecurity variables could not be assessed in the models. Future research should focus on gaining a more comprehensive understanding of these relationships.

In the present study, region was included as a potential confounder in the analyses but was not statistically significant in the models. While variation within regions exists, with local clusters of higher disease prevalence and greater cattle densities, the study was limited to southern regions where systematic differences in management practices and support systems are less pronounced. This may explain the limited influence of region observed in our models.

### 4.5 Limitations

Our study had a high response rate (79%) and the majority of the respondents answered a wide range of the questions, resulting in a high overall response across the survey. Some limitations should, however, be acknowledged. Respondents in this study might have been more engaged with animal health and disease management than the broader dairy farmer population, as participation in the *M. bovis* project and completion of the questionnaire likely required an interest in these issues. It should be noted that only around 15% of the farms agreed to participate in the overall *M. bovis* project (28). Farmers affiliated to the national biosecurity program may have been more motivated and engaged in biosecurity measures which could introduce bias in the observed association between *M. bovis* negative farms and biosecurity program affiliation. Additionally, survey-based studies are susceptible to response bias, where answers may not fully reflect actual practices. Verification of responses was not possible in this study. Future studies should consider validating selected practices through independent observations or records to reduce over- or under-reporting of socially desirable management practices and in that way improve data accuracy. Furthermore, the cross-sectional design of this study limits causal inference, and observed associations may reflect management responses to infection rather than risk factors contributing to its introduction or spread. These limitations should be considered when interpreting the findings. However, there is no indication that any potential bias would be systematic in a way that compromises the comparison between farms with positive or negative *M. bovis* status.

## 5 Conclusion

This study identified farm-level management practices associated with *M. bovis* antibody status. The results suggest that particular attention should be paid to increase the affiliation to the national biosecurity program, since this was associated with antibody negative status. Although identifying management factors is a first step in understanding risk factors for *M. bovis* infection, more research is needed to understand the causal relationships. Disseminating this information to Swedish dairy farmers and their advisors could enhance disease control strategies and help mitigate the spread of *M. bovis* in cattle populations.

## Data Availability

The original contributions presented in the study are included in the article/Supplementary material, further inquiries can be directed to the corresponding author.
