# Supplementary material for: Management and biosecurity practices associated with Mycoplasma bovis seropositivity in Swedish dairy herds: a questionnaire study
Source: Front Vet Sci. 2025 Sep 9;12:1652374. doi: 10.3389/fvets.2025.1652374 (PMC12456186; doi:10.3389/fvets.2025.1652374)
Supplement: Supplementary file 1 [file Supplementary_file_1.docx]

Supplementary Material

# Supplementary material S1. Questionnaire

## Questions about the respondent

1. * Write the herd’s ID number:
2. Name of the person answering the survey:
3. Main job title of the person answering the survey:
   - Farm owner
   - Foreman
   - Animal caretaker
   - Other, name what

## Questions about Mycoplasma bovis

Now, a few questions about *Mycoplasma bovis*, a bacterium that primarily causes mastitis in cows, pneumonia, arthritis, and/or otitis media in calves and young stock. The infections are difficult to treat and often become chronic.

1. During the last 12 months: Have anything of the following been noticed in the herd? (you may tick several boxes)
   - Cows with mastitis did not improve with penicillin treatment. (For example, if the udder was not restored, or if the cow was sent for slaughter or euthanasia due to the udder).
   - Calves with pneumonia that did not improve with penicillin treatment (Penovet, Ethacillin). (For example, calves that needed to be euthanized or had a change in antibiotics).
   - Animals that have suffered from a drooping ear or have walked with their head tilted (inflammation of the middle ear), and there may also be discharge from the ear.

| Cows with mastitis |  |
| --- | --- |
| Calves with pneumonia |  |
| Drooping ear |  |
| Number of answers |  |

1. Have Mycoplasma bovis been diagnosed in the herd?
   - Yes
   - No
   - Don’t know
2. When was the diagnosis Mycoplasma bovis confirmed for the first time?
   - Year:
   - Month:
3. At what kind of testing was Mycoplasma bovis diagnosed?
   - Milk sample from individual cow
   - Nasal swab from calf or young stock
   - Bulk tank milk sample
   - Autopsy
   - Other sampling, what kind?

## Questions about biosecurity and animal contacts

1. Have you ever bought, rented or borrowed cattle into your herd during the last THREE years?
   - No
   - Yes
2. Have you ever bought, rented or borrowed cattle into your herd during the last 12 months?
   - No
   - Yes
3. The cattle introduced to the herd in the last 12 months have been (you may tick several boxes):
   - Breeding bull
   - Lactating cows
   - Heifers for recruitment
   - Cattle for fattening to slaughter
4. Do you use external contract rearing for your heifers?
   - Yes
   - No
5. Do your animals have contact with animals from other herds at the external contract rearing?
   - Yes
   - No
6. How many new animals have been introduced to the herd in the last 12 months (not including own heifers from contract rearing)?
   - None
   - 1 – 2
   - 3 – 5
   - > 5
7. How many different herds did the new animals come from?
   - 1
   - 2 – 4
   - > 4
8. Do you keep new animals separately (stable/building/outdoors) at arrival, without contact with the other cattle at the herd?
   - Always/most of the time
   - Sometimes
   - Rarely/never
9. How long do you usually keep the new animals separately?
   - At least 4 weeks
   - 2 – 4 weeks
   - Less than 2 weeks
10. Are the purchased animals cared for separately from the herd's other animals, with a different animal keeper (alternatively hand washing + change of protective clothing), other machines and separate equipment?
    - Yes
    - No
    - Not applicable, use separate pasture
11. Are the separate space and its equipment thoroughly emptied and washed (fertilization, pressure washing, disinfection) before any new cattle are entered?
    - Always/most of the time
    - Sometimes
    - Rarely/never
12. Have your animals interacted with cattle from other herds in the last 12 months?
    - Yes
    - No
13. In the last FIVE years - Have you brought any cattle into your herd from another country?
    - Yes
    - No

## Questions about biosecurity – people contacts

1. How many people regularly work with the care of the animals or with tasks in the animals' immediate environment?
   - 1 – 2
   - 3 – 5
   - > 5
2. Do any of these people also care for cattle in other herds?
   - Yes
   - Sometimes
   - No
3. Are there routines for how all cattle handlers should behave (wash, change clothes, etc.) after staying in other herds and after traveling abroad, before rejoining your herd?
   - Yes
   - Yes, partly
   - No
4. Is there at least one set of clean protective clothing and boots for visitors to borrow?
   - Yes
   - No
5. Do professional visitors use the farm's own protective clothing and boots? (Professional = those who also visit other farms on the same day)
   - Always/most of the time
   - Sometimes
   - Rarely/never

## Questions about breeding

1. Does the herd use artificial insemination or own bull?
   - Only artificial insemination
   - Only own bull
   - Both artificial insemination and own bull
2. Who takes care of artificial insemination on the farm? (you can tick several boxes)
   - Farm staff
   - Cattle technician
   - Staff from a farm nearby
3. From which breeding companies are bull doses purchased? (you can tick several boxes)
   - VikingGenetics/ Växa Sverige
   - Semex
   - HB Genetics
   - CRI Sweden
   - GGI Sweden
   - Other company, which one:
4. Have embryos from other herds been transferred to recipient animals on the farm?
   - Yes
   - No

## Questions about the management of the cattle at the farm

### Calves

1. Do you have a person on the farm who is responsible for the care of the calves?
   - Yes
   - No
2. How long is the calf kept with the cow (usually)?
   - The calf is removed from the cow as soon as possible
   - Maximum 12 hours
   - 12 – 24 hours
   - 1 to 4 days
   - More than 4 days
3. How soon is the first colostrum given to most of the newborn calves?
   - Within 2 hours after birth
   - Within 6 hours after birth
   - Don’t know
4. What type of colostrum is mostly given to calves?
   - Fresh milk from the calf’s own mother
   - Frozen quality tested colostrum from another cow
   - Frozen NOT quality tested colostrum from another cow
   - Other, what?
5. How often is the colostrum's antibody content (quality) tested?
   - Always/most of the time
   - Sometimes
   - Never
6. How is the colostrum tested?
   - Brix refractometer
   - Colostrometer
   - Other, what?
7. What type of milk do calves usually get after the colostrum period? (multiple answers are possible)
   - Milk replacer (powder)
   - Transition milk (milk from the second to eighth milking after calving)
   - Whole milk from the bulk tank
   - Milk with high somatic cell count
   - Milk from cows with ongoing antibiotic treatment
   - Milk from cows with withdrawal time after antibiotic treatment
8. Is the milk pasteurized before it is given to the calves?
   - Yes, always
   - Yes, sometimes
   - No
9. How are the calves housed during the milking period? (you can tick several boxes)
   - Individual box
   - Group box
   - Individual calf hut outside
   - Group calf hut outside
   - Other, what?
10. At what age are the calves moved from a single pen to a group box during the milking period?
    - < one week of age
    - 1 – 3 weeks
    - > 3 weeks
    - Not moved
11. How are calves usually fed in the group box?
    - Teat bottle
    - Calf feeder with ad libitum milk access
    - Automatic calf feeder
    - Nursing cows
    - Bottle
    - Open tray
    - Other, what?
12. Do the calves that go in a single box/single hut have their own personal teat bucket/pail for milk?
    - Yes
    - No
13. Individual BOX: How often is the milk feeding equipment cleaned? (cleaning means washing with warm water and a brush, possibly washing-up liquid)
    - Every day
    - Several times per week
    - Once a week
    - At arrival of new calves
14. Individual BOX - Where are the calves housed during the milk feeding period?
    - Separate calf stable (calves = animals younger than 6 months)
    - Together with the milking cows
    - Together with the dry cows
    - Together with calving cows
    - Together with young stock
    - Outside
    - Other, what?
15. Group box - Where are the calves housed during the milking period (you can tick several boxes)?
    - Separate calf stable (calves = animals younger than 6 months)
    - Together with the milking cows
    - Together with the dry cows
    - Together with calving cows
    - Together with young stock
    - Outside
    - Other, what?
16. Group box during the milking period - how many calves are there in each group?
    - 2 (pair housing)
    - 3 – 8
    - 9 – 15
    - > 15
17. Is batch rearing used during the milking period, i.e. the group box is completely emptied before new calves are introduced?
    - Yes
    - No
18. Is a sick box used for isolation of sick calves?
    - Yes most of the time
    - Yes sometimes
    - No
19. At what age are bull calves sold (usually)?
    - Before weaning
    - Weaned
    - Not applicable, bull calves are not sold
20. How many weaned calves are there in each group?
    - 2 (pair housing)
    - 3 – 8
    - 9 – 15
    - > 15
21. How are the calf groups for weaned calves separated?
    - Whole walls (no nose contact between different groups)
    - Gates or other more open barriers (nose contact between groups is possible)
    - Different buildings
    - Calf huts
    - Other, what?
22. Where are weaned calves (younger than 6 months) placed (you can check multiple boxes)?
    - Separate calf stable (calves = animals younger than 6 months)
    - Together with the milking cows
    - Together with the dry cows
    - Together with calving cows
    - Together with young stock
    - Outside

***Internal transfer*** *refers to moving from one housing system to another or from one group size to another, such as from a calving pen to a single box, to a group box for milk-fed calves, to a group box for weaned calves, and then to a youngstock barn = 4 times.*

1. How many times is a calf (0-6 months) moved internally within the herd?
   - 1 – 2
   - 3 – 4
   - 5 – 6
   - 7 or more
2. Is surplus feed from the cows' feeding tables used to feed calves and/or young stock?

Calves (0 – 6 months)

- - - Yes
    - No

Young stock

- - - Yes
    - No

### Lactating cows

1. Which housing system is used for the majority of milking cows?
   - Tie stall
   - Loose housing with milking parlour
   - Loose housing with automatic milking
   - Carousel
   - Other, name what?
2. Enter the average number of milking cows in the herd over the last 12 months:
3. Is grouping by udder health used?
   - Yes/always
   - Sometimes
   - Rarely/never
4. Do you use gloves when milking the cows?
   - Always/usually
   - Sometimes
   - Rarely/never
   - Not applicable, the cows are milked in automatic system

*For example: Calving pen – Group of freshly calved cows – Early lactation – Late lactation – Early single group – Group for cows close to calving. Answer = 5 times.*

1. How many times is a milking cow moved during a lactation?
   - 1 – 2
   - 3 – 4
   - 5 – 6
   - 7 or more
2. Where does the calving take place (usually)?
   - Individual calving pen
   - Group calving pen
   - At cow cubicle (tie stall)
   - Other place, where?
3. If group calving pen is used, how many cows are usually kept together?
   - 2 – 4
   - 5 or more
4. Are the same individual boxes used for calving cows and sick cows?
   - Always/usually
   - Sometimes
   - Rarely/never

### Hygiene and health monitoring at the farm

1. What percentage of cows have been treated for mastitis in the last 12 months?
   - 0 – 5 %
   - 5 – 10 %
   - 10 – 15 %
   - 15 – 20 %
   - > 20 %
   - Or enter the number of treated cows during the last 12 months:
2. What percentage of calves in the age 0 – 2 months have had diarrhea the last 12 months? (indicate roughly)
   - None/very few
   - Around one fourth (25 %)
   - Around half (50 %)
   - More than half (50 %)
   - Or enter the number of calves (0-2 months) with diarrhea the last 12 months:
3. What proportion of calves in the age group 0 - 6 months have been treated with antibiotics or antiinflammatory (NSAIDs) due to respiratory disease/pneumonia in the last 12 months? (indicate roughly)
   - None/very few
   - Around one fourth (25 %)
   - Around half (50 %)
   - More than half (50 %)
   - Or enter the number of calves treated for respiratory disease/pneumonia the last 12 months:
4. The last 12 months: How many animals have been treated and/or euthanized due to arthritis? (indicate roughly)
   - The number of calves (0 – 6 months):
   - The number of young stock (> 6 months):
   - The number of cows:
5. How many calves (0-6 months) have died or been euthanized in the last 12 months? (not stillborn)
   - The number of calves:
   - Or calf mortality in percentage (%):
6. Other comments for the entire survey. Please write here:

# Supplementary Tables

| **Table S2**. Distribution of potential risk factors associated with having a positive or negative   *M. Bovis* status for 115 Swedish dairy herds. | | | | |  |
| --- | --- | --- | --- | --- | --- |
| Variable | Category | *M. Bovis* status | | P-value |  |
|  |  | Negative  herds | Positive  herds |  |  |
| New animals are kept separately | Always | 29 | 15 | 0.595 |  |
|  | Sometimes | 16 | 7 |  |  |
|  | Never | 20 | 11 |  |  |
|  | Not applicable | 14 | 3 |  |  |
| Interaction on pasture with other herds | No | 71 | 31 | 0.684 |  |
|  | Yes | 7 | 4 |  |  |
| Number of persons working with the care of the animals | 1 to 2 | 12 | 6 | 0.424 |  |
|  | 3 to 5 | 51 | 19 |  |  |
|  | >5 | 16 | 11 |  |  |
| Employees are also working in other farms | No | 47 | 24 | 0.22 |  |
|  | Sometimes | 22 | 5 |  |  |
|  | Yes | 10 | 7 |  |  |
| Breed^1^ | SRB | 12 | 2 | 0.253 |  |
|  | SH | 28 | 17 |  |  |
|  | Other | 27 | 12 |  |  |
| Routines for rejoing the herd after travelling abroad or visiting other farms | No | 21 | 9 | 0.858 |  |
|  | Yes | 58 | 27 |  |  |
| Professional visitors use the farm's own protective clothing and boots | Yes | 49 | 26 | 0.558 |  |
|  | Sometimes | 20 | 7 |  |  |
|  | Never | 10 | 3 |  |  |
| Breeding strategy | Only AI | 59 | 20 | 0.374 |  |
|  | AI and/or natural service | 24 | 12 |  |  |
| AI^2^ is performed by | Farm staff | 54 | 23 | 0.547 |  |
|  | Technician | 10 | 7 |  |  |
|  | Both | 15 | 5 |  |  |
| Bull doses are purchased from | Viking genetics | 48 | 16 | 0.495 |  |
|  | Semex | 6 | 4 |  |  |
|  | Viking genetics and Semex | 7 | 5 |  |  |
|  | Mix or other | 16 | 9 |  |  |
| Embryo transfer from other herds | No | 61 | 31 | 0.269 |  |
|  | Yes | 18 | 5 |  |  |
| Person responsible for caring of calves | No | 27 | 12 | 0.929 |  |
|  | Yes | 52 | 24 |  |  |
| Colostrum is given to calves | <6h post partum | 53 | 21 | 0.363 |  |
|  | <2h post partum | 26 | 15 |  |  |
| Type of colostrum | Fresh milk from the mother | 67 | 27 | 0.207 |  |
|  | Defrosted milk from other cow | 12 | 9 |  |  |
| Way of testing the colostrum quality | Brix refractometer | 28 | 10 | 0.543 |  |
|  | Colostrometer | 21 | 13 |  |  |
|  | Not tested | 30 | 13 |  |  |
| Transition milk fed to calves | No | 37 | 19 | 0.596 |  |
|  | Yes | 41 | 17 |  |  |
| Fresh milk to calves | No | 50 | 21 | 0.555 |  |
|  | Yes | 28 | 15 |  |  |
| Milk with high SCC^3^ to calves | No | 46 | 25 | 0.284 |  |
|  | Yes | 32 | 11 |  |  |
| Milk from cows on antibiotic treatment is fed to calves | No | 70 | 30 | 0.332 |  |
|  | Yes | 8 | 6 |  |  |
| Withdrawal milk to calves | No | 48 | 26 | 0.267 |  |
|  | Yes | 30 | 10 |  |  |
| Pasteurization of milk before feeding it to calves | No | 69 | 30 | 0.682 |  |
|  | Yes | 9 | 5 |  |  |
| Housing of calves during milk feeding period | Combination of individual and group pens | 41 | 16 | 0.682 |  |
|  | Group pens only | 7 | 2 |  |  |
|  | Individual pens only | 10 | 3 |  |  |
|  | Outside | 7 | 5 |  |  |
|  | Both inside and outside | 14 | 9 |  |  |
| Age of calves when moved from single pen to group pen during milk feeding period | >3 weeks | 22 | 10 | 0.271 |  |
|  | 1 to 3 weeks | 40 | 23 |  |  |
|  | < 1 weeks | 10 | 3 |  |  |
|  | Not moved | 6 | 0 |  |  |
| Feeding method for calves in group pens | Teat bucket | 45 | 15 | 0.251 |  |
|  | Automatic calf feeder | 14 | 12 |  |  |
|  | Bucket | 12 | 5 |  |  |
|  | Other | 6 | 4 |  |  |
| Cleaning of milk feeding equipment for individual pens | Every day | 39 | 15 | 0.797 |  |
|  | Several times a week | 8 | 5 |  |  |
|  | Once a week | 24 | 12 |  |  |
|  | For a new calves | 6 | 4 |  |  |
| Cleaning of milk feeding equipment for group pens | Every day | 39 | 14 | 0.222 |  |
|  | Several times a week | 12 | 4 |  |  |
|  | Once a week | 20 | 14 |  |  |
|  | For a new calves | 3 | 4 |  |  |
| Housing of calves during milk feeding period | Separate calf barn | 30 | 17 | 0.786 |  |
|  | Together with lactating cows | 25 | 9 |  |  |
|  | With dry cows, fresh cows and/or youngstock | 15 | 21 |  |  |
|  | Outside | 7 | 11 |  |  |
| Batch rearing of calves | No | 24 | 8 | 0.233 |  |
| during the milk feeding period |  |  |  |  |  |
|  | Yes | 48 | 28 |  |  |
| Time when bull calves are sold | Before weaning | 28 | 14 | 0.633 |  |
|  | After weaning | 29 | 10 |  |  |
|  | Not applicable | 22 | 12 |  |  |
| Contact between weaned calf groups | No | 18 | 10 | 0.563 |  |
|  | Yes, nose contact | 61 | 26 |  |  |
| Number of times a calf is moved within the herd | 1 to 2 | 28 | 14 | 0.722 |  |
|  | >3 | 51 | 22 |  |  |
| Surplus feed from cows is used to feed youngstock | No | 52 | 21 | 0.439 |  |
|  | Yes | 27 | 15 |  |  |
| Housing and milking system for lactating cows | Loose-housed with AMS | 41 | 20 | 0.523 |  |
|  | Loose-housed with parlour | 29 | 9 |  |  |
|  | Loose-housed with rotary | 5 | 4 |  |  |
|  | Tie stall | 4 | 3 |  |  |
| Use of gloves when milking | Always | 34 | 15 | 0.743 |  |
|  | Sometimes | 10 | 3 |  |  |
|  | Not applicable | 35 | 18 |  |  |
| Number of moves for a cow during a lactation | 1 to 2 | 45 | 33 | 0.531 |  |
|  | >3 | 23 | 13 |  |  |
| Place for calvings | Individual calving pen | 42 | 15 | 0.509 |  |
|  | Group calving pen | 31 | 18 |  |  |
|  | Cubicle or outdoors | 6 | 3 |  |  |
| Number of cows per   group calving pen | 2 to 4 | 14 | 10 | 0.437 |  |
|  | >5 | 15 | 7 |  |  |
|  | Not applicable | 50 | 19 |  |  |
| Use of pens for both calvings and sick animals | No | 45 | 18 | 0.487 |  |
|  | Yes | 34 | 18 |  |  |
| Average milk production   (kg ECM)^4^ |  | 10 997 (SD=1177) | 11 143  (SD=1147) | 0.562 |  |

^1^ SR = >80% Swedish red; SH = >80% Swedish Holstein; Other = all other herds

^2^ AI = artificial insemination

^3^ SCC = somatic cell count

^4^ ECM = Energy-corrected milk for the milk recording year 2019/2020

| **Table S3.** Final complete-case model for internal risk factors associated with having a positive *M. Bovis* status (n=36) compared with being a herd with a negative *M. Bovis* status (n=78). | | | | |
| --- | --- | --- | --- | --- |
| Variable | Odds ratio | SE^1^ | P-value | 95% CI^2^ |
| Herd size^3,4^ | 1.78 | 0.75 | 0.17 | 0.78 ; 4.08 |
| Region^4^ |  |  |  |  |
| Skåne | Referent |  |  |  |
| Halland | 0.49 | 0.37 | 0.35 | 0.11; 2.17 |
| Kalmar/Kronoberg | 1.07 | 0.86 | 0.93 | 0.22; 5.17 |
| Västergötland | 0.49 | 0.31 | 0.26 | 0.14; 1.68 |
| Östergötland | 0.81 | 0.63 | 0.79 | 0.18; 3.70 |
| Use of milk replacer | |  |  |  |
| No | Referent |  |  |  |
| Yes | 3.55 | 1.83 | 0.01 | 1.29; 9.75 |
| Group size of weaned calves | |  |  |  |
| 3 to 8 | Referent |  |  |  |
| 9 to 15 | 0.78 | 0.44 | 0.66 | 0.26; 2.37 |
| >15 | 5.96 | 4.55 | 0.02 | 1.33; 26.66 |
| Intercept | 0.02 | 0.03 |  |  |

^1^ SE= Standard error

^2^ CI = confidence interval

^3^ Log-transformed average herd size

^4^ Included as confounder

**
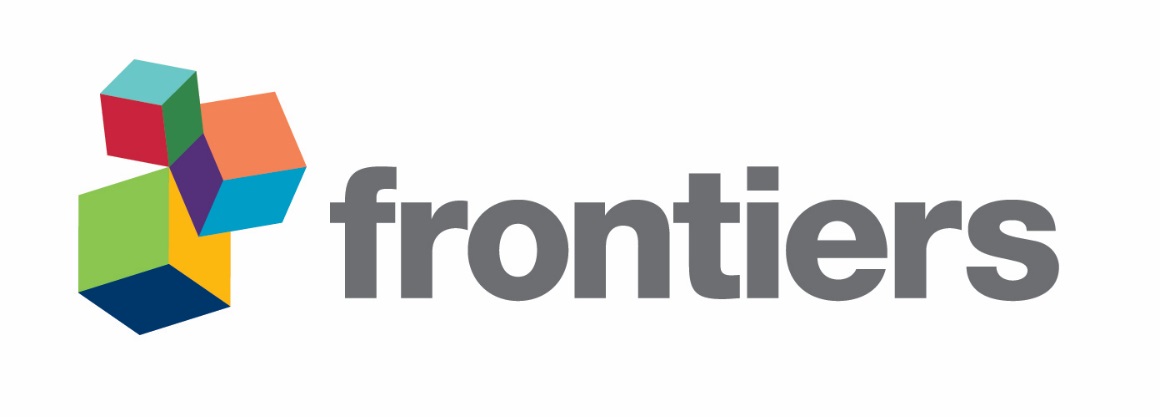
**
